# Establishment and validation of a prognostic risk classification for patients with stage T1-3N0M0 esophageal squamous cell carcinoma

**DOI:** 10.1186/s13019-023-02294-2

**Published:** 2023-06-14

**Authors:** Yang-Yu Huang, Yan Zheng, Shen-Hua Liang, Lei-Lei Wu, Xuan Liu, Wen-Qun Xing, Guo-Wei Ma

**Affiliations:** 1 The Department of Thoracic Surgery, Sun Yat-Sen University Cancer Center, State Key Laboratory of Oncology in South China, Collaborative Innovation Center for Cancer Medicine, Sun Yat-Sen University, No. 651 Dongfengdong Road, Guangzhou, 510060 People’s Republic of China; 2 The Affiliated Cancer Hospital of Zhengzhou University/Henan Cancer Hospital, No. 1 Jianshedong Road, Zhengzhou, 45000 People’s Republic of China; 3 Department of Thoracic Surgery, Shanghai Pulmonary Hospital, Tongji University School of Medicine, Shanghai, People’s Republic of China; 4 Faculty of Biology, Medicine and Health, School of Biological Sciences, The University of Manchester, Manchester, UK

**Keywords:** Esophageal squamous cell carcinoma, Prognostic model, Survival, Stage T1-3N0M0, Surgery

## Abstract

**Introduction:**

At present, clinical factors and hematological indicators have been proved to have great potential in predicting the prognosis of cancer patients, and no one has combined these two valuable indicators to establish a prognostic model for esophageal squamous cell carcinoma (ESCC) patients with stage T1-3N0M0 after R0 resection. To verify, we aimed to combine these potential indicators to establish a prognostic model.

**Methods:**

Stage T1-3N0M0 ESCC patients from two cancer centers (including training cohort: N = 819, and an external validation cohort: N = 177)—who had undergone esophagectomy in 1995–2015 were included. We integrated significant risk factors for death events by multivariable logistic regression methods and applied them to the training cohort to build Esorisk. The parsimonious aggregate Esorisk score was calculated for each patient; the training set was divided into three prognostic risk classes according to the 33rd and 66th percentiles of the Esorisk score. The association of Esorisk with cancer-specific survival (CSS) was assessed using Cox regression analyses.

**Results:**

The Esorisk model was: [10 + 0.023 × age + 0.517 × drinking history − 0.012 × hemoglobin–0.042 × albumin − 0.032 × lymph nodes]. Patients were grouped into three classes—Class A (5.14–7.26, low risk), Class B (7.27–7.70, middle risk), and Class C (7.71–9.29, high risk). In the training group, five-year CSS decreased across the categories (A: 63%; B: 52%; C: 30%, Log-rank *P* < 0.001). Similar findings were observed in the validation group. Additionally, Cox regression analysis showed that Esorisk aggregate score remained significantly associated with CSS in the training cohort and validation cohort after adjusting for other confounders.

**Conclusions:**

We combined the data of two large clinical centers, and comprehensively considered their valuable clinical factors and hematological indicators, established and verified a new prognostic risk classification that can predict CSS of stage T1-3N0M0 ESCC patients.

## Introduction

Esophageal carcinoma (EC) is one of the most prevalent malignancies worldwide and its cause is multi-factorial. The incidence and mortality of esophageal malignancies rank ninth and sixth, respectively, in the global cancer spectrum [1]. About 509,000 mortalities caused by these carcinomas were reported in 2018 alone [1]. Although EC is particularly prevalent in China, Japan, South Africa, Uruguay, France, and Italy [2], more than half of the newly diagnosed EC cases occur in China, resulting in the highest mortality rate in this country.

The major histological subtypes of EC include esophageal squamous cell carcinoma (ESCC) and esophageal adenocarcinoma, with ESCC being the most common [3], accounting for more than 90% of EC cases [4–6]. While research has shown that neoadjuvant therapy can improve the prognosis of patients with ESCC who underwent esophagectomy, unfortunately the postoperative prognosis for these patients remains poor. Specifically, in China, the postoperative 5-year survival rate is only 20–40% [7, 8]. In order to improve the overall survival in patients with EC, it is necessary to identify those patients with a high risk of cancer recurrence and metastasis as early as possible. An accurate staging system and individualized prognosis prediction could help clinicians to identify patients with poor prognoses correctly [9]; this is essential for ensuring such patients receive a multi-disciplinary treatment regimen to improve the curative effects of treatment on EC. Existing research has identified specific factors that directly influence the occurrence and progress of EC, including the preoperative C-reactive protein/albumin ratio [10], neutrophil-to-lymphocyte ratio [11], and thoracic lymph node metastasis [12], among other factors [13–18]. However, currently, there are no methods that can accurately predict postoperative prognosis in patients with stage T1-3N0M0 ESCC.

To address this issue, we developed a prognostic risk classification (referred to as Esorisk) that was based on five different parameters, including the patient’s age, drinking history, number of removed lymph nodes, preoperative hemoglobin level, and preoperative level of albumin. We used patients’ data from Sun Yat-sen University Cancer Center (SYSUCC, training cohort) and that from the Affiliated Cancer Hospital of Zhengzhou University/Henan Cancer Hospital (HNCH, validation cohort). Following this, we used the risk score generated by Esorisk to categorize the cohort into low-risk, middle-risk, and high-risk subgroups that were associated with survival outcomes. This approach provided us with a clinically applicable tool that may assist clinicians with treatment recommendations in this heterogeneous patient subgroup.

## Materials and methods

### Patients

**Fig. 1:**
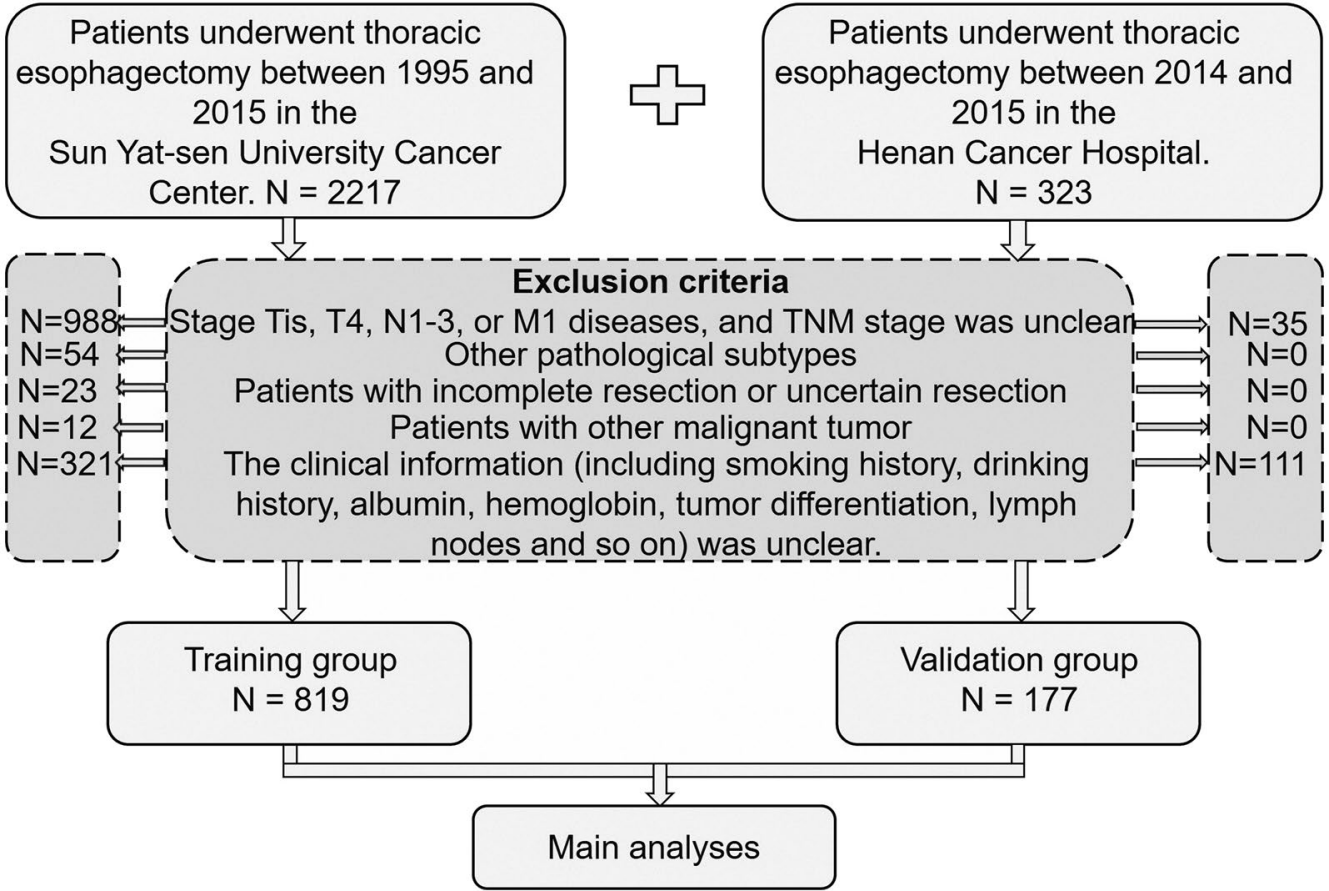
The flow chart of this study

The Ethics Committee of SYSUCC approved the study’s protocol (approval number: YB2016-070). The study data was recorded at the Research Data Deposit of SYSUCC for future reference, with approval number: RDDB2019000777. A total of 996 patients (819 patients from SYSUCC; 177 patients from HNCH) who underwent esophagectomy at the Department of Thoracic Surgery of SYSUCC (Guangzhou, China) or HNCH (Zhengzhou, China) between April 1995 and December 2015 were enrolled retrospectively in the present study. Patients eligible for this cohort study had pathologically confirmed stage T1-3N0M0 ESCC according to the 8^th^ edition of the American Joint Committee on Cancer Staging Manual. The data obtained from SYSUCC were considered the training group. The data from HNCH were considered the external validation group. The flow chart of the study is shown in Fig. 1.

### Surgery

Several standard surgical approaches were used, including the Sweet (left thoracotomy and diaphragm incision), McKeown (right thoracotomy, laparotomy, and neck incision), and Ivor Lewis (laparotomy and right thoracotomy) procedures. In all patients, thoracoabdominal lymphadenectomy was performed.

### Follow-up

We recommended that the patients visited the outpatient department for a follow-up examination every 3–6 months for the first 2 years, every 6 months for the next 3 years, and then every year after that. Follow-up examinations consisted of history assessment, barium esophagography, physical examination, chest radiography, cervical ultrasonography, abdominal ultrasonography, and neck-abdomen computed tomography. If necessary, patients underwent positron emission tomography-computed tomography, endoscopy, or both.

### Statistical analysis

Statistical analysis was performed using SPSS Statistics 25.0 software (IBM SPSS, Inc., Armonk, IL, USA) and R version 3.6.2 (https://www.r-project.org/). The odds ratios with 95% confidence intervals (95% CIs) were calculated by multivariable logistic regression analyses. Hazard ratios (HRs) with 95% CIs were calculated by univariable and multivariable Cox regression analyses. Multivariable analysis was performed to evaluate the effect of sex, age, number of lymph nodes removed, smoking history, drinking history, surgical approach, transthoracic laterality, pathological tumor (pT) stage, tumor grade, preoperative albumin level, and preoperative hemoglobin level on cancer-specific survival (CSS). Standard deviations were used to assess the stability of data. Variables in the univariable analysis that achieved a *P* value of < 0.05 were entered in the multivariable analysis. The most valuable prognostic factors were further confirmed by multivariable analysis. In addition, Kaplan–Meier (K–M) analyses and log-rank tests were used to compare survival curves between different groups. Cases were censored either at patient death or at the end of the follow-up. The selection of CSS as a primary clinical endpoint was considered the most clinically relevant.

In the present study, patient demographics and clinical characteristics were reported for the training cohort. The Esorisk scoring system for CSS was constructed using the linear predictor of the logistic model, which was derived from the training data set. The selected variables were those that achieved a *P* value of < 0.05 in the multivariable analysis or variables considered to be related to death events. The factors used to construct the model were not related to pathological status. The cohort was categorized into Class A (low risk), Class B (middle risk), and Class C (high risk) based on the 33rd and 66th percentiles among the risk scores in the training cohort, and a risk score cutoff was defined for classifying patients in the validation cohorts. Esorisk was applied to calculate the risk scores in the validation cohort, and patient discretization into low-risk, middle-risk, and high-risk subgroups was done using the same cutoffs defined in the training data set. A two-sided *P* value of < 0.05 was considered reflective of statistical significance.

## Results

### Patient characteristics

The clinical characteristics of the patients from the SYSUCC and HNCH databases are listed in Table 1. Among the 996 patients, 680 (68.3%) patients were men, and 316 (31.7%) were women. The patients’ ages ranged from 28 to 88 years (median, 59 years). The 1-, 3-, and 4-year CSS rates were 92.0%, 73.0%, and 49.0%, respectively, and the mean time from surgery to the last censoring date was 71.42 months. During the operation, the median number of lymph nodes dissected were 18.3 ± 13.0 and 17.6 ± 9.2 in the SYSUCC and HNCH cohorts, respectively. There was no pathological diagnosis of lymph node metastasis. None of the patients had received adjuvant or neoadjuvant cytotoxic chemotherapy, radiotherapy, or immune checkpoint inhibitors. According to the patients’ records (including results of computed tomography and operation records), we accurately identified the pathological staging according to the 8th edition of the American Joint Committee on Cancer Staging Manual. The median follow-up time was 75.0 and 57.0 months for the SYSUCC and HNCH cohorts, respectively.

**Table 1:** The associations of clinicopathological characteristics between training cohort (SYSUCC) and external validation cohort (HNCH)

Variables	All patients (N = 996)	Training Cohort (SYSUCC, N = 819)	Validation Cohort (HNCH, N = 177)
	No. of patients/ mean ± std		
Sex			
Male	680 (68.3%)	577 (70.5%)	103 (58.2%)
Female	316 (31.7%)	242 (29.5%)	74 (41.8%)
Smoking history			
No	416 (41.8%)	318 (38.8%)	98 (55.4%)
Yes	580 (58.2%)	501 (51.2%)	79 (44.6%)
Drinking history			
No	703 (70.6%)	590 (72.0%)	113 (63.8%)
Yes	293 (29.4%)	229 (28.0%)	64 (36.2%)
Tumor differentiation			
Grade I	300 (30.1%)	226 (27.6%)	74 (41.8%)
Grade II	474 (47.6%)	388 (47.4%)	86 (48.6%)
Grade III	222 (22.3%)	205 (25.0%)	17 (9.6%)
T stage			
T1	187 (18.8%)	125 (15.3%)	62 (35.0%)
T2	287 (28.8%)	244 (29.8%)	43 (24.3%)
T3	522 (52.4%)	450 (54.9%)	72 (40.7%)
Surgical approach			
Sweet	NA	538 (65.7%)	NA
Ivor-Lewis	NA	23 (2.8%)	NA
McKeown	NA	129 (23.1%)	NA
Other	NA	69 (8.4%)	NA
Age (year)	59.3 ± 9.08	58.4 ± 9.24	63.5 ± 6.92
Hemoglobin (g/L)	135.6 ± 14.59	136.6 ± 14.39	130.6 ± 14.54
Albumin (g/L)	43.20 ± 3.89	43.16 ± 3.94	43.06 ± 3.69
Lymph nodes	18.15 ± 12.50	18.3 ± 13.0	17.6 ± 9.2
Transthoracic laterality			
Left	648 (65.1%)	538 (65.7%)	110 (62.1%)
Right	279 (28.0%)	212 (25.9%)	67 (37.9%)
Other	69 (6.9%)	69 (8.4%)	0 (0%)

SYSUCC, Sun Yat-sen University Cancer Center; HNCH, Henan Cancer Hospital; std, Standard deviations

### Construction of Esorisk

**Fig. 2:**
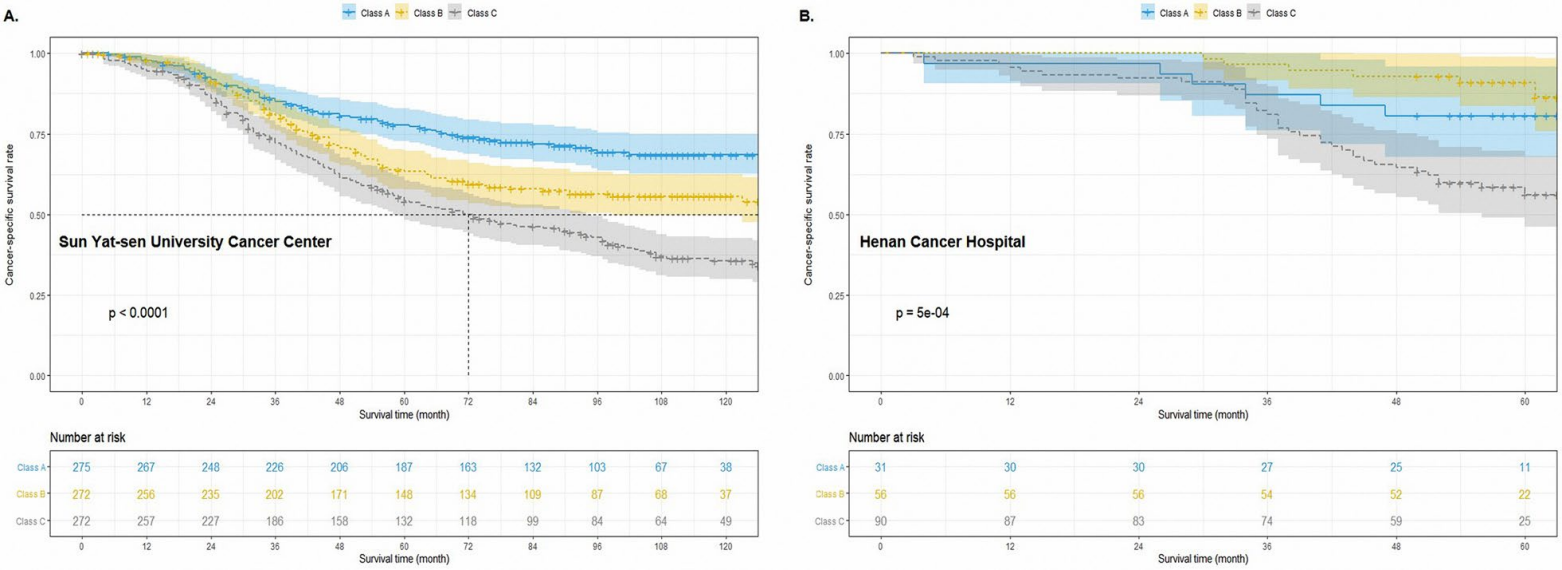
Application of Esorisk to refine the risk assessment in patients with stage T1-3N0M0 esophageal squamous cell cancer in two cohorts. **A** The training cohort. **B** The external validation cohort

As shown in Table 2, multivariable analyses identified the following five clinical characteristics as significant death-dependent factors in patients with ESCC: age, preoperative albumin level, drinking history, number of lymph nodes removed, and preoperative hemoglobin level. Based on the results from the training group, we constructed the Esorisk classification and tested the covariates listed in Table 2 for their association with death events. The parsimonious Esorisk model was as follows: 10 + 0.023 × age + 0.517 × drinking history − 0.012 × preoperative hemoglobin level − 0.042 × preoperative albumin level − 0.032 × number of lymph nodes removed. We did not include factors related to the pathological status in this model. We then used the risk score generated from the above model to classify the cohort into Class A (5.14–7.26, low risk), Class B (7.27–7.70, middle risk), and Class C (7.71–9.29, high risk) depending on the 33rd (7.26) and 66th (7.70) percentiles of the Esorisk score. In the training group, the 5-year CSS decreased across the classes (A: 63%, B: 52%, C: 30%; log-rank *P* < 0.001; Fig. 2A). The results of the Cox regression analysis with adjustment for pT stage, tumor differentiation, and other patient-related factors revealed that the Esorisk class remained significantly associated with CSS (Class B: HR 1.544, 95% CI 1.129–2.111; Class C: HR 2.416, 95% CI 1.748–3.339; *P* < 0.001; Table 3).

**Table 2:** The results of multivariable Logistic regression analysis in ESCC patients with stage T1-T3N0M0 (dependent variable: death events)

Covariate	Coefficient estimate	Odds ratio	95% Confidence interval	*P* value
Age	0.023	1.023	1.007–1.040	0.006
Drinking history				
No vs. yes	0.517	1.676	1.194–2.353	0.003
Hemoglobin (g/L)	− 0.012	0.988	0.977–0.999	0.038
Albumin (g/L)	− 0.042	0.958	0.922–0.996	0.034
Sex				
Female vs. male	0.163	1.177	0.817–1.695	0.382
Surgical approach				0.506
Sweet		1	reference	
Ivor-Lewis	0.604	1.830	0.756–4.425	0.180
McKeown	− 0.022	0.979	0.657–1.457	0.915
Other	− 0.172	0.842	0.489–1.449	0.535
Lymph nodes	− 0.032	0.969	0.955–0.982	< 0.001

ESCC, esophageal squamous cell carcinoma

Age, hemoglobin, albumin and lymph nodes were numeric variables

The method was “Enter selection”

**Table 3:** Univariable and multivariable Cox regression of stage T1-T3N0M0 ESCC patients in the Sun Yat-sen University Cancer Center

Covariate	Univariable Cox regression		Multivariable Cox regression	
	*P* value	HR (95% CI)	*P* value	HR (95% CI)
Sex				
Female		1 (reference)		1 (reference)
Male	0.899	1.016 (0.799–1.292)	0.197	1.177 (0.919–1.507)
Age (continuous)	0.008	1.016 (1.004–1.028)	0.972	1.000 (0.987–1.013)
Smoking history				
No		1 (reference)		
Yes	0.084	1.220 (0.973–1.529)		
pT stage	0.012		0.001	
T1		1 (reference)		1 (reference)
T2	0.05	1.457 (0.999–2.125)	0.04	1.490 (1.019–2.177)
T3	0.003	1.692 (1.191–2.405)	< 0.001	1.935 (1.354–2.766)
Tumor differentiation	0.006		< 0.001	
Grade I		1 (reference)		1 (reference)
Grade II	0.947	1.009 (0.776–1.312)	0.413	1.117 (0.857–1.457)
Grade III	0.007	1.479 (1.111–1.969)	< 0.001	1.763 (1.314–2.366)
Esorisk class	< 0.001		< 0.001	
Class A		1 (reference)		1 (reference)
Class B	0.010	1.477 (1.100–1.984)	0.007	1.544 (1.129–2.111)
Class C	< 0.001	2.316 (1.762–3.044)	< 0.001	2.416 (1.748–3.339)
Surgical approach	0.001		0.014	
Sweet		1 (reference)		1 (reference)
Ivor-Lewis	0.041	1.793 (1.025–3.138)	0.014	2.152 (1.68–3.964)
McKeown	0.027	0.727 (0.548–0.965)	NA	NA
Other	0.014	1.615 (1.103–2.366)	NA	NA
Transthoracic laterality	0.007		0.121	
Left		1 (reference)		1 (reference)
Right	0.120	0.812 (0.625–1.056)	0.802	0.962 (0.714–1.298)
Other	0.014	1.613 (1.101–2.363)	0.046	1.485 (1.007–2.191)

ESCC, esophageal squamous cell carcinoma; CI, confidence interval; The method was “Enter selection”

### Validation of Esorisk

In order to validate the predictive accuracy of the Esorisk classification for CSS in ESCC patients with stage T1-3N0M0, we tested the Esorisk model independently in the validation cohort of 177 patients from HNCH. Using the same risk score cutoff values as those identified in the training cohort allowed us to stratify the patients within the validation cohort into the low-, middle-, and high-risk subgroups. Notably, the high-risk subgroup had a significantly lower 5-year CSS than did the low-risk and middle-risk subgroups (Class A: 81%, Class B: 69%, Class C: 48%; *P*˂0.001; Fig. 2B). The results of the Cox regression analysis with adjustment for pT stage, tumor differentiation, and other patient-related factors revealed that the Esorisk class remained significantly associated with CSS (Class C: HR 3.113, 95% CI 1.225–7.914, *P* = 0.019, Table 4).

**Table 4:** Multivariable Cox regression of stage T1-T3N0M0 ESCC patients in the Henan Cancer Hospital

Covariate	Multivariable Cox regression	
	*P* value	HR (95% CI)
Sex		
Female		1 (reference)
Male	0.714	1.113 (0.627–1.977)
Age (continuous)	0.849	1.004 (0.961–1.050)
pT stage	< 0.001	
T1		1 (reference)
T2	0.343	1.580 (0.614–4.064)
T3	< 0.001	4.411 (2.068–9.412)
Tumor differentiation	0.885	
Grade I		1 (reference)
Grade II	0.873	1.048 (0.589–1.863)
Grade III	0.686	0.798 (0.267–2.387)
Esorisk class	0.001	
Class A		1 (reference)
Class B	0.699	0.802 (0.262–2.455)
Class C	0.019	3.113 (1.225–7.914)
Transthoracic laterality		
Left		1 (reference)
Right	0.343	1.329 (0.739–2.391)

ESCC, esophageal squamous cell carcinoma; CI, confidence interval; The method was “Enter selection”

### Subgroup analysis

**Fig. 3:**
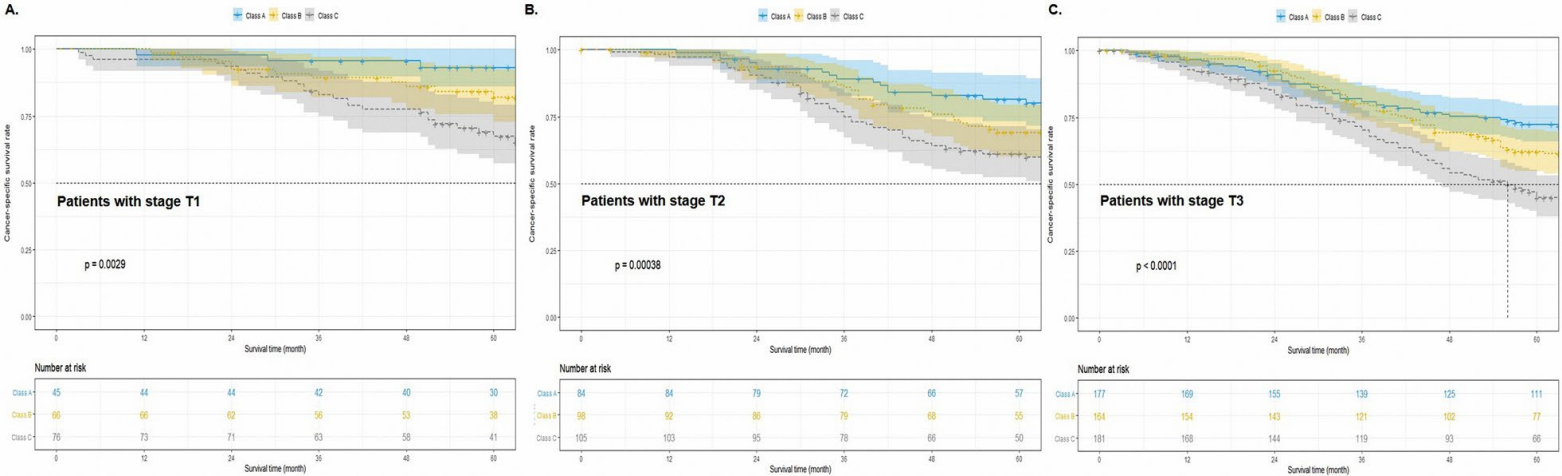
Application of Esorisk to refine the risk assessment in patients with esophageal squamous cell cancer in different stages. These included stage T1N0M0 (**A**), stage T2N0M0 (**B**), and stage T3N0M0 (**C**)

To further explore the effects of Esorisk stratification in patients with ESCC in different T stages, we used K-M analyses to draw survival curves. As expected, the results showed that the Esorisk classification could identify the cohort with poor prognosis among patients with ESCC in every T stage (all *P* < 0.05, Fig. 3).

## Discussion

It is well known that ESCC is associated with poor prognosis, with a 5-year overall survival rate of about 20–40%. The research focus is increasingly being directed at developing better treatments and improving the prognosis for patients diagnosed with ESCC. Previous studies suggested that certain factors have an effect on the prognosis of patients with ESCC; however, a systematic analysis of these factors as prognostic tools has not been done [19–24]. In the present study, we analyzed the data of patients with ESCC from two academic institutes: SYSUCC and HNCH. We considered the data from SYSUCC as the training group, which was analyzed by applying multivariable logistic regression and univariable and multivariable Cox regression analyses. We identified the five factors of age, preoperative albumin level, drinking history, number of lymph nodes removed, and preoperative hemoglobin level as independent factors related to death events. To construct the Esorisk model, we performed calculations using the data related to these five significant variables from patients in the training cohort (Table 2). We regarded the risk score generated by the Esorisk model as the prognostic score of patients. Our results revealed that Esorisk could stratify the patients by their prognosis, and we could identify the patient subgroup with poor prognosis after esophagectomy (Fig. 2, all *P* < 0.001). To validate Esorisk, we used the same method and cutoff values from the training cohort in the external validation cohort. As expected, the validation cohort was similarly stratified by their prognosis (Fig. 2B). Together, the findings from this study support that our established and validated Esorisk model can predict the CSS for patients with stage T1-3N0M0 ESCC, which may help clinicians screen subgroups to identify patients with poor prognoses. Further exploration of the stratification power of the Esorisk classification for patients in different pT stages showed that Esorisk had good discriminatory power in every T stage (all *P* < 0.05, Fig. 3).

The selected clinical factors could be easily obtained from patients’ medical records. Indeed, we were able to retrieve information on the patients’ ages at the time of diagnosis from patients’ medical records and obtain additional data on tumor differentiation, pT stage, and number of lymph nodes removed from patients’ pathological reports. Preoperative levels of albumin and hemoglobin were extracted from patients’ laboratory test results. The easy attainability of these five factors from patients’ records was beneficial for testing our model. In addition, the areas with a high incidence of EC in China are mainly located in Henan Province, Hebei Province, the Chaoshan region of Guangdong Province, and other regions [25–27]. SYSUCC is located far from HNCH, and both cancer centers accept patients with ESCC across China. Our model was constructed using cases from SYSUCC and further validated using cases from HNCH. We excluded patients with stage T4 ESCC and those with metastases in lymph nodes and other organs because a previous study showed that neoadjuvant therapy could improve the prognosis in patients who were diagnosed with metastases in lymph nodes before surgery [28].

Some limitations to our study should be noted. First, the sample size of patients with ESCC was insufficient; the T stage was restricted to only T1-3, and the data distribution of the T stage was not balanced. To improve this aspect, the sample size would need to be expanded in further studies. Second, the patients’ data used in this study originated from two academic institutes only, which might also affect the accuracy of our model. While the results presented herein indicated that our five-factor prognostic model was useful for predicting the CSS for patients T1-3N0M0 ESCC postoperatively, a larger, multi-center study will be required to verify the applicability of this model. Third, this model only provides certain reference information to clinicians, not the treatment recommendations. The doctors would need to make treatment-related decisions according to the relevant guidelines and their clinical experience. Fourth, the follow-up period for the HNCH cohort was short. More follow-up research is needed to evaluate the prognosis for these patients. Fifth, the patients’ records lacked the results of molecular diagnoses, such as information on the expression of programmed death-ligand 1, which is an effective prognostic factor that could improve prediction accuracy [29–31]. Therefore, substantial research at the molecular level is needed to enhance the proposed model.

## Conclusions

In conclusion, we established and validated a novel prognostic risk classification that can predict the CSS for patients with T1-3N0M0 ESCC. This valuable information may help clinicians detect subgroups of patients with poor prognoses. Moreover, we think that this model, based on patient data from two academic institutes, could easily be applied by other researchers to their own data.

## Data Availability

Data from this study are available from the corresponding author upon reasonable request. The clinical data has been uploaded in the Research Data Deposit (approval number: RDDA20200001368; http://www.researchdata.org.cn/).

## Abbreviations

ESCC: Esophageal squamous cell carcinoma
CSS: Cancer-specific survival
EC: Esophageal carcinoma
SYSUCC: Sun Yat-sen University Cancer Center
HNCH: Henan Cancer Hospital
HRs: Hazard ratios
pT: Pathological tumor
K-M: Kaplan–Meier
CI: Confidence interval
